# Income level and treatment selection in prostate cancer: analysis of a North Carolina population-based cohort

**DOI:** 10.1093/jncics/pkad032

**Published:** 2023-04-27

**Authors:** Crosby Rock, Ying Cao, Aaron J Katz, Deborah Usinger, Sarah Walden, Ronald C Chen, Xinglei Shen

**Affiliations:** Department of Radiation Oncology, University of Kansas Medical Center, Kansas City, KS, USA; Department of Radiation Oncology, University of Kansas Medical Center, Kansas City, KS, USA; Department of Population Health, University of Kansas Medical Center, Kansas City, KS, USA; Department of Radiation Oncology, Lineberger Comprehensive Cancer Center, University of North Carolina at Chapel Hill, Chapel Hill, NC, USA; Department of Radiation Oncology, Lineberger Comprehensive Cancer Center, University of North Carolina at Chapel Hill, Chapel Hill, NC, USA; Department of Radiation Oncology, University of Kansas Medical Center, Kansas City, KS, USA; Department of Radiation Oncology, University of Kansas Medical Center, Kansas City, KS, USA

## Abstract

**Background:**

Disparities in treatment selection based on socioeconomic status for prostate cancer exist. However, the association between patient-level income with treatment selection priorities and treatment received has not been studied.

**Methods:**

A population-based cohort of 1382 individuals with newly diagnosed prostate cancer was enrolled throughout North Carolina prior to treatment. Patients self-reported household income and were asked about the importance of 12 factors contributing to their treatment decision-making process. Diagnosis details and primary treatment received were abstracted from medical records and cancer registry data.

**Results:**

Patients with lower income were diagnosed with more advanced disease (*P* < .01). Cure was deemed to be “very important” by more than 90% of patients at all income levels. However, patients with lower vs higher household income were more likely to rate factors beyond cure as “very important” such as cost (*P* < .01), effect on daily activities (*P* = .01), duration of treatment (*P* < .01), recovery time (*P* < .01), and burden on family and friends (*P* < .01). On multivariable analysis, high vs low income was associated with increased utilization of radical prostatectomy (odds ratio = 2.01, 95% confidence interval = 1.33 to 3.04; *P* < .01) and decreased use of radiotherapy (odds ratio = 0.48, 95% confidence interval = 0.31 to 0.75; *P* < .01).

**Conclusions:**

New insights from this study on the association between income and treatment decision-making priorities provide potential avenues for future interventions to reduce disparities in cancer care.

Prostate cancer is the most frequently diagnosed nondermatologic cancer among men, with an estimated 268 000 new diagnoses in the United States in 2022 (1). The vast majority of patients present with curable disease (2) and face a choice of selecting from multiple treatment options including radiation therapy (RT), radical prostatectomy, and active surveillance (3-7). Long-term outcomes are generally favorable for patients with prostate cancer; however, disparities exist. Socioeconomic status (SES), a broad concept that incorporates education, employment, and income, may influence treatment selection and outcomes in patients with prostate cancer. Lower SES has been associated with lower rates of curative treatment (8-10) and higher rates of prostate cancer mortality (8,9,11,12).

However, prior studies have mostly used area-level indicators as a surrogate for patient SES (10-13), and therefore, the impact of patient SES on prostate cancer care remains understudied. The present study fills this current knowledge gap using data collected from a prospective, population-based cohort that collected detailed information on patient-level SES measures, treatment decision-making factors, and treatment received. We hypothesized that, on an individual level, patients with lower income have worse prostate cancer characteristics at diagnosis and different goals of care and make different treatment decisions compared with patients with higher income.

## Methods

### Patient population

The North Carolina Prostate Cancer Comparative Effectiveness and Survivorship Study is a population-based cohort of patients newly diagnosed with prostate cancer between 2011 and 2013. Details regarding survey design, patient identification, and enrollment have been previously described (14). Briefly, patients were identified at the time of their prostate cancer diagnosis through the Rapid Case Ascertainment program of the North Carolina Central Cancer Registry with all 100 North Carolina counties participating. Potential enrollees provided written informed consent and were contacted via telephone for enrollment and survey administration. Each survey was conducted by a trained interviewer. All patients were enrolled prior to receiving prostate cancer treatment and followed prospectively. The survey enrolled 1456 participants of whom 1382 reported household income and comprise the current study population. This study was approved by the University of North Carolina at Chapel Hill institutional review board.

### Data collection

Information concerning prostate cancer diagnosis (Gleason score, prostate-specific antigen [PSA] level, cancer stage) was obtained from the North Carolina Central Cancer Registry; these data elements were used to classify patients into prostate cancer risk groups per National Comprehensive Cancer Network guidelines (7). Medical records were collected to determine primary treatment modality received within the first 12 months following diagnosis. Individual-level sociodemographic information including date of birth, race, marital status, educational level, health insurance status, and household income was collected by patient report at baseline. Urban and rural residence were categorized using the US Department of Agriculture Rural-Urban Continuum Code (RUCC) of the county of primary residence, with RUCC 1-3 categorized as urban and RUCC 4-9 as rural.

Cohort participants were surveyed regarding factors relevant to their treatment decision-making process. Participants were asked to rate each of the following factors as “very important,” “somewhat important,” or “not important” when choosing a treatment option: cancer cure, preserving quality of life, preserving sexual function, ability to control urination, preserving bowel function, being a burden on family and friends, cost of treatment, duration of treatment, recovery time after treatment, treatment impact on daily activities, following doctor’s recommendations, and following recommendations from family and friends. Participants were further asked to rate the single most important factor among cancer cure, preserving quality of life, being a burden on family and friends, cost of treatment, treatment impact on daily activities, and other. Although no validated questionnaire exists to assess prostate cancer treatment decision-making factors, these questions were created based on feedback from patient stakeholders who helped design the North Carolina Prostate Cancer Comparative Effectiveness and Survivorship Study.

### Statistical analysis

Descriptive statistics were used to summarize patient characteristics, treatments received, and patient-reported decision factors. We compared differences across income groups using the χ^2^ test. Multivariable logistic regression models examined factors associated with receipt of radical prostatectomy as primary treatment; the final model included income level, age, prostate cancer risk group, urban or rural residence, race, education, employment, and insurance. A separate model examined factors associated with RT. A *P* value of less than .05 was considered statistically significant. All statistical analyses were performed using SAS 9.4 (SAS Institute, Inc, Cary, NC, USA).

## Results

Among 1382 cohort patients, 539 (39%), 553 (40%), and 290 (21%) reported low ($<40K), medium ($40-90K), and high ($≥90K) household income, respectively (Table 1). Household income was associated with race, educational attainment, and health insurance status—demonstrating that these individual-level sociodemographic measures are correlated.

**Table 1.** Baseline patient characteristics by income group^a^

Characteristics	Low income (n = 539)	Intermediate income (n = 553)	High income (n = 290)	*P*
Median age (IQR), y	65 (60-70)	65 (59-69)	62 (56-67)	<.01
Race, No. (%)				<.01
Black	239 (44.3)	110 (19.9)	28 (9.7)	
White	271 (50.3)	434 (78.5)	259 (89.3)	
Other	29 (5.4)	9 (1.6)	3 (1.0)	
Education, No. (%)				<.01
8th grade or less	43 (8.0)	6 (1.1)	1 (0.3)	
Some high school	262 (48.6)	145 (26.2)	14 (4.8)	
Some college or more	234 (43.4)	402 (72.7)	275 (94.8)	
Insurance, No. (%)				<.01
Uninsured	40 (7.4)	8 (1.4)	0 (0)	
Medicaid	78 (14.5)	13 (2.3)	3 (1.0)	
Medicare	307 (57.0)	281 (50.8)	103 (35.6)	
Private	114 (21.1)	251 (45.5)	184 (63.4)	
Employed, No. (%)	128 (23.8)	253 (45.8)	207 (71.4)	<.01
Residence, No. (%)				
Urban	396 (73.5)	401 (72.5)	234 (80.7)	.03
Rural	143 (26.5)	152 (27.5)	56 (19.3)	
Gleason score, No. (%)				.14
≤6	289 (53.6)	337 (61.0)	175 (60.3)	
7	196 (36.4)	182 (32.9)	95 (32.8)	
8-10	54 (10.0)	34 (6.1)	20 (6.9)	
PSA, median (IQR)	6.1 (4.8-8.7)	5.4 (4.4-7.4)	4.6 (3.8-6.3)	<.01
Risk group, No. (%)				<.01
Low risk	284 (52.7)	335 (60.6)	174 (60.0)	
Favorable intermediate risk	66 (12.2)	62 (11.2)	50 (17.2)	
Unfavorable intermediate risk	128 (23.7)	119 (21.5)	45 (15.5)	
High risk	53 (9.8)	34 (6.2)	20 (6.9)	
Metastatic	8 (1.5)	3 (0.5)	1 (0.3)	

a IQR = interquartile range; PSA = prostate-specific antigen.

Patients with lower income were more likely to present with more advanced disease at diagnosis, including higher PSA levels (*P* < .01) and higher risk group (*P* < .01) (Table 1).

To gain further insight into treatment selection, we analyzed data on patient-reported treatment decision-making factors (Table 2). Although more than 90% of patients in all 3 income groups rated cure as “very important,” differences were observed across groups in many other factors. Treatment cost was deemed “very important” by 61.2%, 38.5%, and 14.5% (*P* < .01) of low, intermediate, and high earners, respectively, whereas duration of treatment was rated “very important” by 58.4%, 42.7%, and 24.5% (*P* < .01), respectively. Additionally, recovery time was considered an important decision-making factor more frequently among lower-income patients, with 68.4% rating it as “very important” compared with 52.3% and 37.8% of intermediate- and high-income earners (*P* < .01), respectively (Table 2).

**Table 2.** Percentage of patients who indicated that each of these treatment decision-making factors was “very important”

Factors	Low income, %	Intermediate income, %	High income, %	*P*
Cure	91.2	90.8	93.3	.78
Cost	61.2	38.5	14.5	<.01
Effect on daily activities	64.4	63.1	60.6	.01
Duration of treatment	58.4	42.7	24.5	<.01
Recovery time	68.4	52.3	37.8	<.01
Burden on family and friends	78.8	76.0	65.2	<.01
Quality of life	84.2	84.8	89.6	.03
Preserved sexual function	51.9	52.7	53.1	<.01
Preserved urinary continence	83.3	83.8	80.9	.33
Preserved bowel function	87.2	85.6	85.5	.20
Doctor recommendation	88.8	82.1	65.6	<.01
Input of family and friends	44.4	30.2	19.1	<.01

The decision-making factor that patients selected as being the single most important consideration also differed by income group (*P* < .01; Table 3). For example, 74.3% of high-income vs only 54.4% of low-income patients indicated “cure” as the most important factor. A higher proportion of patients in the low-income group selected burden on friends and family, cost, and effect on daily activities as being most important.

**Table 3.** Decision-making factor patients selected as the single most important consideration in choosing treatment, by income group

Factors	Low income, %	Intermediate income, %	High income, %	*P*
				<.01
Cure	54.4	61.9	74.3	
Quality of life	28.8	29.3	23.7	
Patient burden on friends and family	9.3	5.2	0.4	
Cost	3.3	1.0	0.4	
Effect on daily activities	3.0	2.1	1.2	
Other	0.5	0.5	0.0	

Primary treatment also differed by income level. In patients with low-risk prostate cancer, low-income patients were more likely to receive RT, whereas high-income patients were more likely to receive radical prostatectomy. In patients with intermediate- and high-risk prostate cancer, low-income patients were more likely to receive no treatment or RT compared with high-income patients (Table 4).

**Table 4.** Initial treatment received by income group

Treatment	Low income	Intermediate income	High income	*P*
Low risk (n = 793), No. (%)				.006
No treatment	118 (41.6)	123 (36.7)	68 (39.1)	
Radical prostatectomy	73 (25.7)	120 (35.8)	67 (38.5)	
Radiation therapy	78 (27.5)	86 (25.7)	31 (17.8)	
Other^a^	15 (5.3)	6 (1.8)	8 (4.6)	
Intermediate and high risk (n = 577), No. (%)				<.001
No treatment	30 (12.2)	19 (8.8)	10 (8.7)	
Radical prostatectomy	82 (33.2)	107 (49.8)	83 (72.2)	
Radiation therapy	110 (44.5)	70 (32.6)	16 (13.9)	
Other^a^	25 (10.1)	19 (8.8)	6 (5.2)	

a Other includes primary hormone therapy, cryotherapy, high-frequency ultrasound, or unspecified other treatment not listed.

On multivariable analyses, adjusting for age, race, prostate cancer risk group, and other patient factors, high income (vs low income) was associated with increased odds of undergoing prostatectomy (odds ratio [OR] = 2.01, 95% confidence interval [CI] = 1.33 to 3.04; *P* < .01) and reduced odds of RT (OR= 0.48, 95% CI = 0.31 to 0.75; *P* < .01) (Table 5). Income appears to be an independent factor for primary treatment selection even after accounting for race.

**Table 5.** Multivariable logistic regression examining factors associated with receipt of radical prostatectomy or radiation therapy as primary treatment^a^

Factors	Radical prostatectomy		Radiation therapy	
	OR (95% CI)	*P*	OR (95% CI)	*P*
Income (reference = <$40K)				
$40-$90K	1.66 (1.20 to 2.30)	<.01	0.83 (0.61 to 1.12)	.23
>$90K	2.01 (1.33 to 3.04)	<.01	0.48 (0.31 to 0.75)	<.01
Age, per year	0.89 (0.87 to 0.91)	<.01	1.03 (1.01 to 1.05)	.01
Risk group (reference = low risk)				
Favorable intermediate	2.42 (1.66 to 3.53)	<.01	1.40 (0.96 to 2.05)	.08
Unfavorable intermediate	2.13 (1.55 to 2.94)	<.01	1.75 (1.29 to 2.39)	<.01
High	3.04 (1.90 to 4.88)	<.01	1.30 (0.82 to 2.08)	.26
Residence (reference = urban)				
Rural	1.09 (0.81 to 1.46)	.56	1.16 (0.87 to 1.55)	.30
Race (reference = White)				
Non-White	0.69 (0.50 to 0.94)	.02	1.30 (0.96 to 1.76)	.09
Education (reference = 8th grade or less)				
High school	1.39 (0.64 to 3.04)	.41	1.18 (0.60 to 2.30)	.63
College	1.36 (0.62 to 2.96)	.44	1.13 (0.58 to 2.21)	.73
Employment (reference = yes)				
No	1.00 (0.73 to 1.36)	.99	1.08 (0.78 to 1.48)	.65
Insurance (reference = private)				
Uninsured	0.59 (0.29 to 1.21)	.15	1.62 (0.81 to 3.22)	.16
Medicaid	1.13 (0.63 to 2.04)	.68	0.99 (0.55 to 1.77)	.97
Medicare	0.96 (0.66 to 1.38)	.82	1.45 (1.00 to 2.10)	.05

a CI = confidence interval; OR = odds ratio.

## Discussion

In this prospective, population-based cohort of 1382 prostate cancer patients, we demonstrated a statistically significant association between lower patient household income and presentation with more advanced disease at time of diagnosis. Furthermore, we found that although patients in all income levels reported cure as the most important decision-making factor, those with lower income were more likely to emphasize cost, duration of treatment, recovery time, burden on family and friends, and effect on daily activities. Lastly, we found that treatment modality varied notably by patient income, with lower-income patients having greater use of RT and less use of radical prostatectomy compared with higher-income patients even after adjusting for other factors including race and prostate cancer risk group.

In agreement with prior literature, we found household income was associated with later stage at diagnosis. This may be partly attributable to lower screening rates among those of lower SES. Rapiti et al. (10) demonstrated reduced rates of PSA screening–detected prostate cancer in lower SES individuals as compared with their high SES counterparts by a margin of 14.8%. Similarly, Gilligan et al. (15) demonstrated a strong negative relationship between poverty (OR = 0.33; *P* < .001) or near poverty (OR = 0.69; *P* < .001) and use of PSA screening. Our study further underscores the importance of improved access to care and prostate cancer screening for those of lower SES as a means by which disparities in oncologic outcomes among varying socioeconomic classes might be mitigated.

To our best knowledge, this is one of few population-based studies to examine patient-level household income with prostate cancer diagnosis and treatment. Although prior studies have consistently shown an association between area-level SES indicators and patient race with prostate cancer treatment (10-13,15-17), the reasons behind this difference have not been well studied. The current study is unique because every patient was enrolled prior to treatment and because of the study’s specific focus on assessing factors important during the patient’s treatment decision-making process. These factors likely help explain treatment differences by income.

All patients valued cure as the most important consideration. However, low-income patients also felt other factors were important as well. Broadly, cost of care, cost of factors associated with treatment such as logistics of treatment, and concern about being a burden to family may be encompassed in the concept of financial toxicity (18,19). Potentially, newer treatment methods that shorten treatment time or have lower cost such as stereotactic body radiotherapy (SBRT) (20) offer more attractive options for these patients. In general, high-income patients had differences in decision-making factors such as decreased importance placed in the opinion of friends, family, and physicians as compared with patients with low income. The reason for this is unclear and merits additional investigation. Our study suggests that patient income should inform approach to treatment counseling and may be incorporated into patient decision-making tools (21).

The current study has several strengths. One important strength is the large cohort size, allowing for statistically meaningful evaluation of all 3 income levels. An additional advantage of the current investigation over previously reported literature evaluating the effect of SES on the prostate cancer experience is the availability of patient-level SES data, including household income. Prior work largely relied on surrogate markers such as occupation (8) and patient geographic area of residence (13,17) to approximate SES. The availability of individual-level SES data allowed us to study the potential impact of SES on patients’ priorities during the treatment decision-making process. An additional strength of this study is the population-based cohort, which uniquely enrolled each patient prior to treatment and is diverse with respect to both race and SES. However, a patient cohort from a single state might limit generalizability of study findings.

The current study has limitations. The North Carolina population may not necessarily be generalizable to the United States. Enrollment in the current study was between 2011 and 2013. Given the long interval since data accrual, there is the potential for changes in practice patterns over the past 10 years. We note that the high-income cohort had a slightly younger median age, and this may be a confounding variable in decision making. We also recognize the strong association between SES and race, which may confound our findings, but note that on multivariate analysis, we observed difference by income even after adjusting for age and race. Lastly, clinical data such as medical comorbidities, a potential cofounding variable, are unavailable making further evaluation not possible.

In conclusion, this population-based cohort of 1382 men with newly diagnosed prostate cancer found that patients with lower vs higher household income were more likely to present with advanced disease at diagnosis, consider factors in addition to cure in their treatment decision-making process, and choose no treatment or RT over radical prostatectomy. Further work must be done to address the disparity in access to appropriate cancer care in low-income patients with prostate cancer.

## Data Availability

Study data underlying this article will be shared on reasonable request to the corresponding author.
